# The impossibility of engaged research: Complicity and accountability between researchers, ‘publics’ and institutions

**DOI:** 10.1111/1467-9566.13418

**Published:** 2021-12-07

**Authors:** Veronica Heney, Branwyn Poleykett

**Affiliations:** Wellcome Centre for Cultures and Environments of Health, University of Exeter, Exeter, UK; Faculty of Social & Behavioural Sciences, University of Amsterdam, Amsterdam, The Netherlands

## Abstract

Over the past decade, U.K. universities have increasingly sought to involve publics in research as active participants in the construction of academic knowledge. Sociologists of health have largely welcomed this enthusiasm for engaged and participatory ways of working, including methodologies long in use in the field such as patient-led research and co-creation. Despite the strong interest in engaged research, however, we argue that funding patterns, bureaucratic structures and an overreliance on people employed on casual contracts make it extremely difficult, often impossible, to do engaged research in British universities. Drawing on our own experiences, we show how our attempts to practise and deepen accountability to variously situated publics were constrained by the way our institution imagined and materially supported engagement. We argue that it falls to individual researchers to mitigate or work around structural barriers to engagement, and that this process creates dilemmas of complicity. If engaged research is to fulfil its remit for inclusion and its radical potential, researchers need to think carefully about how the U.K. engagement agenda entwines with processes of casualisation, acceleration and projectification, and how institutional recuperations of engagement can undermine its political and epistemic objectives.

## Introduction

Researchers in British universities are increasingly expected to engage nonacademic publics in academic knowledge production. Publics are no longer seen as subjects of research, or as audiences for research dissemination; they are invited into academic spaces to co-create knowledge. Increasing engagement with research is intended to achieve a wide range of outcomes: from improving public scientific literacy, demonstrating value for money and creating more “socially useful” knowledge, to destabilising, even dismantling, hierarchies of academic knowledge production (Martin, 2008; Rose & Kalathil, 2019; Stevens et al., 2014). Including publics in research is particularly urgent in the context of public health research where doing research in collaboration with, and not “on”, most affected people are seen to contribute to renewing and repairing research relationships that have been previously experienced as exploitative (Hunt, 1981; Reynolds & Sariola, 2018). British researchers navigate a specific public engagement agenda promoted by U.K. Research Councils (UKRI, 2021). In the 2000s, “public engagement” was directed primarily at STEM subjects and “premised on a model of dialogic interplay between nonexpert public groups and academic experts” (Watermeyer, 2011). Engagement, however, has significantly expanded to encompass an ethos of partnership and co-creation in research, and as it has done so, it has both resonated, but also conflicted, with longstanding projects of creating counter-hegemonic knowledge inside and outside of the academy.

Both of us responded to calls for funding, which specifically sought out "engaged research" projects, and we were encouraged to either design our projects from scratch using engaged methodologies, or reorient our existing research to meet new criteria. Engaged research as practised at the institution where we worked together is intended to depart from existing “public engagement” strategies designed to “share” the benefits of higher education and research with the public (National Coordinating Centre for Public Engagement, 2021), following criticism of such endeavours as formalising and solidifying “deficit-led approaches” (Hinchliffe et al., 2018: 4) in which researchers are positioned as experts who possess knowledge, and “the public” is understood as lacking in knowledge. We take engaged research to mean the range of practices through which research is embedded in communities from the outset, not through “outreach” or “consultation” but through continuous co-creation, where the social goods of research in the form of remuneration, data, cultural capital and access to decision makers are generated in participation with communities and, ideally, equitably shared.

Public health research has a long tradition of using different forms of engagement (including public and patient involvement (PPI) and co-creation) to reach marginalised people who in the past have been excluded or had their experiences distorted or misrepresented. Engaged research frameworks draw on a range of concepts, values and techniques of research: translation, equity, justice, fairness, transparency and co-construction. As engaged researchers, we explicitly understand engaged research as drawing on pre-existing traditions of feminist and activist research, as well as PPI, user-led and participatory approaches. The sociology of health and medicine is strongly influenced by PPI, which has long been a vital framework (Maguire & Britten, 2018), emerging out of a strong activist tradition around health-care and disability rights encapsulated by the slogan ‘Nothing About Us Without Us’ (Charlton, 1998). Yet, subsequent to the institutionalisation of PPI following the Department of Health's INVOLVE initiative (Rose, 2014: 151), distinctions have been drawn between more tokenistic, consumerist approaches such as upstream consultation and what is considered “authentic” or transformative involvement and co-production (Madden & Speed, 2017; Williams et al., 2020).

Thus, we might also see as significant a focus on ‘user-led’ or ‘survivor’ research, in which those who might be taken as the ‘objects’ of research not only collaborate on its production but also exert control over the research process at all levels (Beresford, 2020; Telford & Faulkner, 2004). Such approaches often explicitly reference participatory research, an approach initially developed in development studies (Chambers, 1983) and described by Bergold and Thomas as oriented towards “planning and conducting the research process with those people whose life-world and meaningful actions are under study” (2012: 4). In other words, there is a rich tradition across these fields of understanding research not as liberation or transformation but as a contingent and complex continuum of more or less constructive practice. These various literatures and practices have long histories and are already in conversation: while we use the term engaged research to describe our work, we see value not in the term's ability to articulate something new but rather in how it expresses a connection to existing fields, recognising this as “a spacious conceptual landscape where multiple terms are used” (Erikainen et al., 2021).

The emergence of the engagement agenda in U.K. universities over the past ten years has coincided with the transformation of these institutions along neoliberal lines and the decline of job security and working conditions across the sector. A range of explanations have been offered for how engagement and engaged research might mesh with other defining features of the contemporary university: acceleration, precarity, competition, elitism and hierarchy. Facer, for example, argues that engagement allows for a measure of access and participation without destabilising hierarchies that underpin knowledge, method and expertise, constituting a “useful inoculation against the potential incursions of unruly publics into the world of increasingly entrepreneurial universities” (Facer, 2020: 22). In this article, we use an analytic of complicity to analyse potential affinities between engaged research and other, parallel ways of assessing, ranking and operationalising research.

The contribution we seek to make to the critical literature on participatory, engaged and co-constructed research is rooted in our experience as early career researchers (at the time of writing, we were a PhD student and a postdoctoral fellow). We begin by considering some of the specific struggles we each experienced as we aligned the particular and context-specific goals of our research with a shared, institutional approach to engaged research. We each explore, from our different perspectives, how we navigated gaps, lapses and silences in how this research was conceived and understood. Heney reflects on researcher positionality in the context of user-led research on mental health and specifically self-harm in the U.K., while Poleykett examines how an emphasis on innovation in engaged research positioned their work on chronicity and everyday in eating in Dakar, Senegal, as ideally exemplifying a U.K.-centric definition of engagement, one that failed to understand histories of community participation and co-production in Senegalese public health. While the disciplinary contexts and geographical locations of our work are different, we both from our specific vantage points consider experiences that became illegible or unspeakable, and experiences and perspectives that went untheorized and unrepresented. In particular, we both consider how specific conceptualisations or mobilisations of engaged research might in fact serve to reproduce, rather than interrupt, ways of doing and valuing research.

We tackle these questions in four subsections: through these different strands and sites of analysis, we hope to demonstrate the impact of institutional structures upon practices of engaged research. We begin by critically reflecting on our experiences of conducting engaged research. In the first section, Heney considers the tendency for engaged research to position direct lived experience of research topics as existing solely outside or beyond the academy. This framing obscures both the complex politics of peer research and the university's undertaking and responsibility to support these researchers. Second, Poleykett examines how conducting engaged research outside of the U.K. showed up how far ideologically, imaginatively and bureaucratically “engagement” was shaped by visions of politics and participation that were highly specific to the U.K. Third, we analyse these experiences in relation to the structural transformation of U.K. higher education over the past decade, tracking the rise and rise of interest in engagement and engaged research alongside parallel processes of acceleration, marketisation, projectification and dependency on precariously employed staff. All of these concomitant trends, we argue, create an environment in which it is very difficult to do engaged research well, even as the expectation that early career scholars will have expertise in engagement is now well established. Focussing specifically on our experiences of practical challenges associated with payment and projectification, we explore how we have sought ways to finesse or otherwise create ‘workarounds’ for institutional limitations. In the fourth and final section, we shift from examining our experiences of navigating these inherent contradictions to examine how the concentration of experimental engaged research practice within cohorts of precarious and early career research is constraining the sustainability and the transformative potential of engaged research. Throughout each of these four strands of analysis, we consider how an attention to institutional contexts allows for exploration of the complex and nuanced forms of complicity generated by attempting to conduct engaged research within existing structures.

## The Blurred Boundaries of Engagement: Doubled Positionality in Public

My (Veronica Heney) work is concerned with cultural representations of self-harm as understood and experienced by people with the experience of self-harm, drawing on critical sociological work on the relevance of social and cultural meanings of self-harm and the inadequacy of medicalised frameworks (Chandler, 2016; Gurung, 2018). Thus, my doctoral research attempts to bring together qualitative sociological methods together with Literary Studies’ textual practices in order to bring the complexities and contradictions of experiences of self-harm to bear on narrative analysis. Here, I explore how my own location within the research project troubled some of the ways in which I found engaged research to be normatively positioned and unaccompanied by structural transformations to the university as a site of labour and knowledge production.

The project not only took shape through my own experiences of self-harm but also has been developed in collaboration with a small group of other people with the experience of self-harm who formed an advisory group and considered issues regarding the design of research questions, the selection of methods, conducting trauma-informed interviews and the accessible dissemination of results. This collaboration was a vital part of my research and of my approach to self-harm as an experience, which requires attention to multiplicity and difference. Yet, I noticed with some mild frustration a tendency within conversations around engaged research to automatically position all researchers as outsiders to the experiences which they researched; as I sat in these conversations, I found myself quietly estranged from my own life and my experience of self-harm elided underneath the totalising label of researcher. It seemed that framings which repeatedly positioned researchers as distant from their fields of study enacted certain assumptions about who is considered capable of carrying out research, and what experiences might already exist within the academy. This positioning is one much critiqued in the literature on insider research. Thus, Villenas has discussed the ways in which monolithic framings of researchers seemed to erase her own experiences of being deeply isolated and uncomfortable within academic classrooms as a Chicana woman (1996) while Dahl calls instead for recognition that “academics and our concepts are always already part of our networks and not outside them” (2010: 165). More specifically, I found my experience echoing Voronka's eloquent exploration of “peer identity” within Mad Studies, and the ways in which the contradictory demands to perform both recognisable madness while still functioning appropriately within professional contexts construct a “crisis of authenticity” (2019: 569).

Yet, I also recognise that my discomfort with this positioning is ambivalent. At times, labelling my research as engaged rather than as user-led or insider research, as indicated by my funding, feels like a relief. It feels like a barrier against enquiries or judgements about my personal life and my personal experiences, which might be painful to me, or which might require me to disclose details I feel incapable of recounting. My concern is less that my own experience might be seen as delegitimising my research (a critique firmly and thoroughly explored in feminist discussions around the standpoint theory (Collins, 1986; Harding, 1992)), but rather that my experience itself might be held up to scrutiny, might be delegitimised and might be dismissed as ‘not enough'. I am certainly not alone in these concerns around authenticity (see Voronka., 2019), which has a particular resonance in the context of self-harm (Scourfield et al., 2011). Through the framework of engaged research, I am instead able to place emphasis on my collaboration with other people with the experience of self-harm and my attempts to ensure the project responds to a multiplicity of perspectives. Of course, I strongly feel that these efforts are both necessary and ethical, and thus perhaps foregrounding them in the framing of the project is appropriate. My experience of self-harm is both singular and privileged, through my position as a white, middle class woman, and as someone who did not experience inpatient care as a result of self-harm. Nevertheless, this also functions as a form of distanciation, allowing me to obfuscate when it feels necessary for my own privacy or comfort. Furthermore, it possibly grants my work as an element of academic legitimacy rather than vulnerability as it locates the project more firmly within traditional scholarly structures and literatures rather than less-valued, community projects.

This distance is possible because of my own privilege; the way in which my particular lived experience and position allows me to access academic spaces and institutions, to be comfortable using academic language and frameworks, and to be read or responded to primarily as a scholar rather than a service user. These privileges allow me to ‘manage my madness’, so to speak, in a context in which, as Margaret Price has laid out at length, the prioritisation of rationality, criticality, presence, participation, productivity, collegiality, security, coherence and independence function to systemically exclude mentally disabled students and researchers (2011). The privacy which I seek or lay claim to (and upon which I sometimes feel dependent) is not evenly distributed or accessed. In allowing myself to feel or make use of its comfort in certain moments, I wonder whether I am failing in the demands of solidarity with those whose race, class, disability or particular ongoing experience of mental distress have barred them from the academic spaces through which I move, or at least from certain protection within those spaces (Rose & Kalathil, 2019).

Moreover, this positioning perhaps allows institutions to claim or push for the cachet of engaged research without fully considering the complexity and even difficulty of embodying and living through the contradictions at the heart of insider or peer research. Thus, it functions to maintain rather than challenging the absence of support and resources for marginalised researchers who are already part of institutions or who might become part of such institutions in the future. If researchers are understood to be reaching out to those who experience research topics, rather than already embodying that experience themselves, then the university is freed from any responsibility to take this into account in providing the working conditions for such research. The university is freed from the following, for instance: providing adequate funding and job security, so that PhDs and research careers are possible for those without generational wealth; from decolonizing institutional practices and spaces and recognising (and repairing) the burden upon researchers of colour of working within the white academy (Bhambra et al., 2018); and from radically reconceptualising ‘access’ so that all staff and students are given the structure and support necessary to work in ways appropriate to them (Minich, 2016). Without such transformations, the potential of engaged research will surely remain unfulfilled, limited to those of us who manage (often through our privilege) to survive within university structures.

## Engagement and Ethnography: National Research Culture and Complicity

I (Branwyn Poleykett) have been conducting long-term, ethnographic research in Dakar, Senegal, for over ten years. My research, which has spanned multiple postdoctoral appointments, is concerned with the practice of public health in the city. Briefly, my current research is concerned with the emergence of chronic disease in Dakar, and how the proliferation of diagnoses of hypertension, diabetes and heart disease is reshaping eating in the city's suburbs. My ethnographic research tacks between the production of authorised, supposedly universal, knowledge about diet and day to day contestation over eating in Dakar's large, multigenerational suburban households. My research examines attempts to adopt, domesticate, translate, live with or otherwise subvert norms of “healthy eating”, not as encounters between naïve publics and novel biomedical knowledge, but as part of ongoing, embodied “situated struggles” for health and wellbeing (Fairhead et al., 2006: 115). I was initially extremely interested in how ethnography could articulate with engaged research methods and approaches.

Having always worked in and through public and global health, I judged myself relatively pragmatic when it came to translating ethnographic data into other disciplinary approaches and conceptual frameworks. In global health, ethnography is often understood and instrumentalised as a form of cultural expertise (Biruk, 2019), a tool for eliciting perspectives and experiences; indeed, it is sometimes taken to be a kind of ‘upstream public engagement’ (Plows, 2008), as much a tool for eliciting and figuring responses as for observing and analysing attitudes and behaviour. Medical anthropologists have written extensively about the complicities that arise from acting as embedded or engaged ethnographers in settings such as clinical trials and public health interventions (Nelson, 2019; Pigg, 2013).

In reflecting on tensions and complicities that emerged from trying to apply engaged research methods to my ongoing ethnographic work, I want to highlight not the conflicts and difficulties I encountered in Senegal in my relationship with collaborators and research participants, but instead the problematic forms of complicity generated “at home” and in relation to the priorities of my home institution. Ultimately, possibilities for reciprocal learning and methodological innovation driven by the significant expertise that existed in Senegal were blocked by a generally weak understanding in the U.K. of the relationship between engagement and place; a strong desire to demonstrate innovation in engaged research and an understanding of engaged research as a U.K. brand with value to offer U.K. institutions.

When studying a diffuse, diffracted and complex process like eating, it is not straightforward to establish access to a ‘public’ united by a common matter of concern, particularly over a question like ‘how shall we eat’, a prompt that often stimulated explosively argumentative responses in Dakar. Nonetheless, I understood that processes of co-creation necessitated the identification of publics tied to particular places and processes. Proximity, place and power are vital questions for engaged research. Engaged research has often worked to weave new relationships of ownership and accountability between universities and their hinterlands, a process that John Holmwood suggests will be vital to universities’ post-COVID futures (2020). Within the university at home, there was no clear sense of how a geographically distant public could be incorporated into a programme of engagement or become the beneficiaries of U.K. research. In continuing to develop collaborations in Senegal, I was made extremely conscious of doing engaged research outside of normal parameters.

Engaged research done outside of the U.K. was often taken to be plugging a gap or making up a deficit. In 2019, for example, I began a new collaborative project with nutritionists, NGO food sovereignty activists and local community members. In our pilot project, we worked with community members from a highly food-challenged context, a cluster of villages on the outskirts of Dakar where community members described the significant food challenges they faced in a context of entrenched poverty and precarious agricultural work. Carrying out this work in close collaboration with a Senegalese NGO, we used techniques that were familiar to me. Before training in academic research, I had worked with the organisation Enda Tiers Monde in Dakar and had absorbed their way of working, a blend of participatory action research and a technique, ethos and practice of solidarity they described as “*accompagnement*”, being with communities in processes of transformation. Described by one of the organisation's founders, Cheikh Hamadou Kane as the “process of understanding and recognition, in the Hegelian sense, of the poor”, Enda's approach has a rich and complex genealogy including the Senegalese tradition of *animation rurale*, a technique of postcolonial pedagogy that encourages critical reflection and transformation.

I was taken aback, then, when I was asked in the U.K. how far my collaborative research was “showcasing our approach”, or when I encountered the assumption that “doing engagement” outside of the U.K. and the global North needed to be carefully documented, so that it was possible to extrapolate learning about how the “unique challenges” of that context could be overcome. Within the university, the question of how to recognise or incorporate a geographically distant “public” could only seem to be conceived of in terms of deficits that public might possess that the application of excellent, U.K.-developed research instruments might mitigate. This misapprehension reflects the highly parochial nature of the U.K. engagement agenda and often weak understanding of how engaged research is envisaged and practiced in other contexts (Hambidge et al., 2019).

Commitments to a place-based engagement, and a limited and ahistorical view of how collectivities are dynamically formed and re-formed independent of the intercession of researchers in this case worked to block mutual learning across disciplinary and methodological boundaries. The significant expertise of my Senegalese collaborators in translating elite knowledge into relevant and digestible communication through techniques of *animation*, and then, in turn, allowing for public contestation to re-work categories of research, was persistently erased in the U.K. where innovation in engaged research was taken to be driven by academics trained in Britain and based on U.K. universities. In this sense, a certain diffuse but intense interest around innovating in engagement blocked possibilities for transnational and reciprocal learning and a fixation of pinning down and exemplifying engagement stymied conversations about what engagement means in other linguistic contexts or communities of practice.

## Engagement and Research in the ‘Accelerated Academy’

We have considered above how we each manage multiple accountabilities within our projects. Research for us has involved becoming accountable, not just to “publics”, but to different histories, methodologies and ways of working. In what follows, we consider how these multiple accountabilities can fracture and be transformed into complicity in the institutional context and structures of U.K. universities and funding bodies. We are not the first to argue that universities and academic institutions often constrain rather than enable co-production and engaged research (Williams et al., 2020). Rose and Kalthil suggest that in relation to mental health, co-production is “likely impossible in privileged sites of knowledge production” such as universities (2019: 1). In what follows, we pay close attention to somewhat mundane and bureaucratic aspects of conducting research, including payment of participants, funding timeframes and job security and precarity. These minutiae are rarely closely examined in policy and briefing documents on engagement but, we argue, decisively shape the enactment of the engagement agenda. Despite claims that engagement is a strategic institutional priority, we lay out tangible aspects of university and funding structures, which make the practices and values of engaged research difficult or impossible to enact, using both specific examples from our experiences and more general observations. We also outline the way in which this might make any engaged research situated in universities complicit in the maintenance of these structures, and that the temptation to find workarounds which allow engaged research to move forward often at the expense of individual researchers might be, despite both good intentions and meaningful or important outcomes, a form of complicity.

It is striking that a broad interest in engaged research has emerged alongside a range of other transformations in U.K. universities, changes that undermine commitments to public accountability. For example, what has been described as the “accelerated academy” (Ylijoki, 2016) sees a shift towards competitive ranking of institutions, an increase in output and monitoring of academic productivity, and new forms of syncopated “project time” in academia as more, and more research takes place within fixed-term teams and consortia, under significant time pressure to demonstrate their impact and turn around publications. This “projectification” of research (Ylijoki, 2016) has a significant impact on engagement, compressing research into discrete blocs and timelines that rarely allow for the continuous, long-term engagement with communities demanded by engaged research. Working in collaboration with public health and along research timeframes set by funders such as the NIHR further compresses and constrains engaged research.

Durie et al. emphasise that “successful engagement projects often require substantial and flexible amounts of time” and suggest that both ‘lead-in’ and ‘follow-on’ periods are essential elements of engagement projects (2011: 5). That this is the case surely becomes obvious from simply a brief consideration of the complexities of establishing relevant contacts, building relationships, agreeing aims and principles among multiple partners, conducting collaborative data analysis or even simply holding meetings in which participants are able to give input into themes and findings, and writing collaboratively or producing work, which contributes to both academic and nonacademic outputs. Complex and perhaps extended timelines are even more likely in engaged projects involving health or illness, in which absence or an altered pace might be particularly likely, especially considering a conceptualisation of crip time as involving “new rhythms, new practices of time and new sociotemporal imaginaries” (Samuels & Freeman, 2021: 251). Yet, funders rarely anticipate or encourage engaged research grants to move more slowly, and in particular, engaged research fellowships and PhDs remain funded for only three years, and standard institutional deadlines for progression and submission remain in place. This, too, places individual researchers in a difficult position in which their individual success or even hope for future employment is dependent on their ability to deliver a project along a timeline, which at times seems utterly incompatible with the tangible practices and political ideals of engaged research.

Certainly, I (Heney) have felt these pressures. For my PhD, I put in a detailed funding application months before I would begin which drew only on my own experience and the relevant literature, I moved across the country and transitioned directly to my PhD from an MA which gave me little time to make preparatory connections, and I was strongly advised to have ethical approval in place before I met formally with collaborators or participants. This meant that by the time my advisory group was formed and meetings began, I was several months into the PhD, the shape of which had already been somewhat delineated both by my application and my early work. Thus, while both the framework and the specific details of my inquiry shifted in several ways as a result of working with my advisory group, the overarching structure of the PhD remains almost entirely my own work. Similarly, I have been consistently aware of the impending end of my funding: not only does engaged work take time, which eats into an inevitably tight PhD timetable, but it also introduces uncertainties and perhaps delays. I worry that my own concern about potential delays led me to tightly delineate which aspects of the project could be discussed or changed, or that my own anxiety about the timeline impacted in more subtle or implicit ways what advisory group members felt comfortable suggesting or criticising. I also note my sense that in attempting to complete a ‘nontraditional’ PhD within a ‘traditional’ timeframe, I would be required to be willing to conduct ‘nontraditional’ elements in ‘my own time’, so to speak, conducting meetings in evenings or at weekends when this was what most convenient for others.

Here, while universities and funding bodies are willing or even proactively seek to fund engaged research, they are less willing to challenge the established systems within which such research must necessarily be carried out. A similar dynamic frequently occurs in relation to paying partners in co-production and engaged research. This is a widely acknowledged frustration in almost all U.K. higher education institutions. Ensuring that all contributors to the research process are adequately compensated is a fundamental aspect of engaged research, reflecting the commitment to valuing equally different (and differently located) forms of expertise (Faulkner, 2004; Rickard & Purtell, 2011). While traditionally research payment has been a contested issue, accepted standards of good practice have broadly shifted even in health-care-related areas, which are regarded as particularly fraught, such as drug studies (Slomka et al., 2007). There can be no equality in research or knowledge production while some parties are paid and others are reduced to the status of volunteers. Moreover, it seems logical that relying upon voluntary labour and thus limiting participation to those who can afford to work for free risks re-entrenching existing barriers to research collaboration along intersecting class and racial lines. This might be agreed in principle, however, there are often many complications in practice: suggested payment amounts are often very low; vouchers are given as a preferred means of payment despite the possibility for them to be experienced as patronising and paternalistic; bank transfers are often possible only after large amounts of time-consuming paperwork and even then are often delayed; cash payments are discouraged and possible only as an exception; and universities are often reluctant to engage with complexities around universal credit or to provide practical or legal support if participating in research leads to claims being denied. Once again, we might locate the increasing institutional interest in engaged research alongside broader social trends, here the context of austerity, a decade of cuts to social security and increasingly punitive welfare systems; this context, too, is of particular relevance to health research and to questions of health inequalities.

Thus, in attempting to act both ethically and in the interests of their partners, collaborators and participants, individual researchers might attempt to find a variety of solutions including investing significant time and energy advocating to members of the university administration for a more appropriate approach, filling out paperwork on behalf of research participants and even paying out of their own pocket regardless of the likelihood of later reimbursement. I (Heney) have adopted a number of these approaches, both in order to make participation in my research as convenient as possible and to recognise that when payment is made complicated or time-consuming, participants might simply opt not to receive it. After many months of advocacy both by myself and by both my supervisors, I was eventually granted an exception and permitted to pay my participants in cash: I was deeply grateful for this, as it allowed me to pay participants without delay and without using my own funds. However, I was very aware of this status as an exception, and that broader university policies remained insensitive to concerns of appropriateness and ease of payment.

Through these two examples, we hope to demonstrate the significance of the bureaucratic contexts within which engaged research exists and is carried out. Engaged research functions not only within systems or hierarchies of knowledge production but also within practical systems, which have vital tangible impacts upon the possibility or impossibility of engaged research. With regards to both project timing and the payment of research partners, researchers often seek individual ‘fixes’ to systemic problems, attempting to hold together through their own willingness to go ‘above and beyond’ a series of incompatible logics, and indeed in their potential success then *uphold* those logics by becoming an exemplar of a way of working. For while, these practices may make possible engaged research (or a sort of engaged research), they lessen or silence the challenge which engaged research seeks to make to structures of power, to the practices of the university and to institutional knowledges. Moreover, they allow those systems to claim their flexibility or their suitability, since universities are able to make use of these projects as case studies and flagship projects without meaningfully addressing the way in which their own structures actively act in opposition to such work, or indeed acknowledging the potential toll which these ‘stop-gaps’ may take on researchers. This dynamic is not uncommon amongst work which attempts to enact criticism or transformation while located within university institutions (Phipps & McDonnell, 2021). This presents a complex dilemma of complicity in which it is difficult for individual researchers to successfully carry out engaged research while simultaneously arguing for the systemic changes and institutional support which is both necessary and urgent.

## Precarity and Inequality in the Academy

Another trend that marks the previous twenty years and runs alongside the rise of the engagement agenda is the increasing precarity of academic employment, a trend which exists in complex relation to engaged research (Ivancheve, 2015; Montoya & Pérez, 2016; Cardozo, 2017). One of the things that we struggled with as junior researchers trying to build progressive methodologies for engaged research was the structure of academic career progression. As in the cognate and adjacent movements in academia towards open access and inter and multidisciplinarity, academic rituals of recognition and attainment appeared to stymie the progress that institutions purported to greatly desire. Indeed, the prevailing tendency within universities seemed to be that the importance of engagement could best be recognised by opening up a “track” or pathway through institutions (Borrow & Russo, 2015; Watermeyer, 2015). When the instability of research careers in relation to public engagement is considered, this is often framed solely as a problem of emotional difficulty or pressure, in which engaged research brings trouble to an otherwise unproblematic working environment (Oliver et al., 2019), rather than adequately contextualised within the already-unsustainable context of current higher education.

As researchers employed on fixed-term contracts, we were increasingly encouraged to see the relationships we had developed with community partners as a kind of fungible asset, a “selling point” for a researcher that she could then transport from position to position and place to place. Setting aside the ethics of instrumentalising trusting, horizontal and collaborative research relationships, trust in these relationships is a precarious and ongoing negotiation that will likely fail if researchers do not have access to the resources to sustain it. As Jones and Oakley argue, “relationships with organisations and communities beyond the university [require] long-term investments and these must therefore be matched by longer-term and secured contracts” (2018: 6). Encouraging engagement work at the lowest rungs of the university hierarchy reflects a broader failure to grapple with the emotional work of building community, particularly in contexts where mistrust of outsiders and the burden of research fatigue is acute. This is certainly likely to be the case for engaged research around health and illness, given historical and ongoing conflicts around unethical or unsuitable research (Hunt, 1981). What also of the potential loss to communities and community structures as researchers and research projects are whisked away at the end of short fixed-term contracts, leaving little chance for follow-up or ongoing contact, as early career researchers are required to devote time and energy to new projects or roles, perhaps in different institutions, locations or even sectors?

We observed the frequency with which people who conduct engaged research were hired at junior levels, while senior or permanent posts are awarded to those with more traditional academic outputs and achievements. We are not interested in the tokenistic promotion of one or two engaged researchers; rather, we would draw attention to a recurring dynamic in which engaged research remains hard to sustain as a result of its predominance primarily among the precariat. Not only does this limit the function and possibilities of individual projects, but also such a dynamic might limit opportunities for widespread systemic change, and the transformation which engaged research claims as its aim and outcome (Hinchliffe, 2018: 4). What is more likely is continued re-invention of the wheel, as waves of junior researchers encounter the impossibilities of engaged research, and become edged out of the academy either through burnout engendered from continually doing battle with these structures or through unending precarity. Engagement cannot become integral to research while it is considered peripheral to the activities that build status and craft reputations and careers.

This broad devaluation of engagement work and its placing, within many universities, on a separate “track” from research, is connected, as Cardozo writes of teaching, to its association with care and relationships and with feminised forms of academic work (Cardozo, 2017). We have observed a gendered pattern of reward and recognition, whereby women are criticised in their research for “not caring enough”, and men receive excessive praise and recognition for experimenting with engagement. Engagement is not only received differently when men and women do it, but it is also deeply related to the unequal ways that universities recruit and retain staff (Bhopal & Pitkin, 2018; Monroe et al., 2008; Shilliam, 2015). Researchers who are women, who are people of colour, who are working class or who have lived the experience of mental and physical illness or disability might be more likely to conduct engaged research projects but will be less likely to find secure academic employment. Indeed, as the first two sections of this paper explored, engaged research often assumes the elite, upper class, white identity (and superiority) not just of academic knowledge, but of academic researchers. Thus, precarity, inequality (in multiple forms) and the devaluation of forms of engaged research are deeply entertwined, perhaps functioning to particularly limit the possibility for sustained work within marginalised communities and by marginalised researchers. This limitation has especially concerning implications within a health-care context in which the health-care inequalities remain a vital issue.

The dislocation between the high value placed on engagement and the lack of serious consideration of how it fits into academic labour and the progression of the academic career has led to the proliferation of unstable and unsustainable projects built on precarious labour. As we near the end of our funded contracts, we feel the difficulties of holding together important research relationships and partnerships in the face of an uncertain future. Our ability to act ethically, to avoid the ‘smash and grab’ research tactics so frequently criticised in public health (Lambert & Carr, 2018: 1276), becomes dependent on our individual professional success in an increasingly competitive and precarious sector. This professional success requires our ability to submit to and meet institutional metrics of achievement which might at times be directly contrary to the aims of engaged research. Each attempt to secure future, usually precarious, employment can feel like a complex form of necessary complicity, not least because we repeatedly ask our partners to invest time and energy into projects and collaborations whose future we cannot guarantee. The broken and exploitative ecosystem of academia impacts not only the lives and careers of individual researchers but spreads outwards to affect research collaborations and to limit what forms of research are truly possible.

## Conclusion

In this paper, we have examined the significant role of institutions in shaping the unfolding of engaged research. Despite the prominent rhetorical support for engagement across research in the U.K., and particularly within health-care research, the structures and practices of universities and funding bodies make engaged research extremely challenging. We connected the current practice of engaged research to broader contexts and transformations within universities, with the acceleration of expectations and workloads, and to the contingent and precarious employment of early career researchers across project work. Complicity provided us with an important analytic to analyse our own experience of working to mediate and mitigate university policies and guidelines and to make participation and engagement possible for our collaborators.

Drawing on difficulties that we encountered trying to align our research with engaged approaches, we examined issues with how our university framed the publics and constituencies of engaged research. In framing these publics as external to the institution and identifying the primary task of research as *incorporating* them, Heney discussed how user-led research and the specific positionalities or experiences of researchers were erased, while Poleykett described how significant expertise in conducting engaged research in other modes and linguistic registers remained invisible. We then explored the difficulty (or impossibility) we experienced in successfully enacting the material demands of engaged research practices, particularly regarding payment and prolonged processes of relationship-building, within the unchanged structures of funding bodies and universities. Finally, we suggested that sector-wide trends of precarity fundamentally limit the sustainability and transformative potential of engaged research. In both instances, we noted the extent to which individual researchers might feel (or indeed be) responsible for resolving the limitations of institutional structures, creating tension around both failures and successes in conducting engaged research.

In particular, we have drawn attention to the tension between the stated desire of universities and funding bodies to fund and develop engaged research, and the existing practices, systems and ways of working within these institutions which make engaged research difficult or impossible. This is a tension which cannot be addressed or resolved by individual researchers or within discrete research projects: it must be meaningfully addressed by institutions, if their stated desire to support engaged research is sincere. To fail to make these necessary changes, leaves researchers carrying out engaged research, particularly those who are precariously employed, who experience systemic marginalisation, or who have lived experience of their research topics, in an impossible and unsustainable position, struggling to navigate often confused and contradictory institutional logics and carry out engaged research.

Through critically reflecting upon both existing literatures and our own experiences, we have sought to convey some of the intersecting textures of complicity which arise through engaged research's location within such structures, recognising and communicating the messy entanglement of accountability, complicity and the engagement agenda. In accounting for this mess and complexity, we have thus attempted to resist what we have identified as an unhelpful trend towards individualisation and thinking in cases. In writing this paper collaboratively and collectively, we were able to both feel the support of shared experience and to think together beyond the individual towards structural forces and institutional contexts through and within which those experiences came to be. Our experiences of navigating engaged research projects resonate beyond an “engagement agenda”. Advocating for institutional support for engaged research offers us possibilities for critique, solidarity and change. Paying attention to and valuing forms of expertise outside of academia requires conversations about collaboration that should not leave intact or take for granted hierarchies and that should explicitly seek to disrupt the smooth functioning of audit-driven academia. Reflecting on engaged research timelines, for example, might give us a lever we can use in collective projects of “pushing back” “against the rhythms of the neoliberal university” (Hughes, 2021). Doing knowledge differently, in partnerships and collaborations that can challenge unethical and elitist research, is not just a question of individual researchers and their capacities, inclinations and commitments. Rather, valuing academic “engagement” requires that we challenge parallel processes of casualisation, projectification and acceleration.

## Supplementary Material

Title Page
