# Supplementary material for: The impossibility of engaged research: Complicity and accountability between researchers, ‘publics’ and institutions
Source: Sociol Health Illn. Author manuscript; Available in PMC 2025 May 5. (PMC7617641; doi:10.1111/1467-9566.13418)
Supplement: Title Page [file EMS204336-supplement-Title_Page.pdf]

Title Page

Title: The impossibility of engaged research: Complicity and accountability between researchers, ‘publics’ and institutions

Author Information:

Veronica Heney

Wellcome Centre for Cultures and Environments of Health, University of Exeter, Exeter, UK

Branwyn Poleykett

b.poleykett@uva.nl

orcid.org/0000-0002-5180-9235

Faculty of Social & Behavioural Sciences, University of Amsterdam, Amsterdam, The Netherlands

Abstract

Over the past decade, U.K. universities have increasingly sought to involve publics in research as active participants in the construction of academic knowledge. Sociologists of health have largely welcomed this enthusiasm for engaged and participatory ways of working, including methodologies long in use in the field such as patient-led research and co-creation. Despite the strong interest in engaged research, however, we argue that funding patterns, bureaucratic structures and an overreliance on people employed on casual contracts make it extremely difficult, often impossible, to do engaged research in British universities. Drawing on our own experiences, we show how our attempts to practise and deepen accountability to variously situated publics were constrained by the way our institution imagined and materially supported engagement. We argue that it falls to individual researchers to mitigate or work around structural barriers to engagement, and that this process creates dilemmas of complicity. If engaged research is to fulfil its remit for inclusion and its radical potential, researchers need to think carefully about how the U.K. engagement agenda entwines with processes of casualisation, acceleration and projectification, and how institutional recuperations of engagement can undermine its political and epistemic objectives.
